# Effect of repeated explicit instructions on visuomotor adaptation and intermanual transfer

**DOI:** 10.1007/s00221-022-06470-z

**Published:** 2022-09-27

**Authors:** Susen Werner, Heiko K. Strüder

**Affiliations:** 1 Institute of Movement and Neurosciences, German Sport University, Am Sportpark Müngersdorf 6, 50933 Cologne, Germany; 2 Institute of Professional Sport Education and Sport Qualifications, German Sport University, Cologne, Germany

**Keywords:** Sensorimotor adaptation, Motor learning, Awareness, Process dissociation procedure, Explicit process, Implicit process

## Abstract

The aim of the present study was to investigate the effect of repeated explicit instructions on visuomotor adaptation, awareness, and intermanual transfer. In a comprehensive study design, 48 participants performed center-out reaching movements before and during exposure to a 60° rotation of visual feedback. Awareness and intermanual transfer were then determined. Twelve participants each were assigned to one of the following adaptation conditions: gradual adaptation, sudden adaptation without instructions, sudden adaptation with a single instruction before adaptation, and sudden adaptation with multiple instructions before and during adaptation. The explicit instructions explained the nature of the visual feedback perturbation and were given using an illustration of a clock face. Analysis of adaptation indices revealed neither increased nor decreased adaptation after repeated instructions compared with a single instruction. In addition, we found significant group differences for the awareness index, with lower awareness after gradual adaptation than after sudden, instructed adaptation. Our data also show increased initial adaptation in aware participants; regardless of whether awareness was developed independently or with instruction. Intermanual transfer did not differ between groups. However, we found a significant correlation between the awareness and intermanual transfer indices. We conclude that the magnitude of the explicit process cannot be further increased by repeated instruction and that intermanual transfer appears to be largely related to the explicit adaptation process.

**Supplementary Information:**

The online version contains supplementary material available at 10.1007/s00221-022-06470-z.

## Introduction

Identifying exercise conditions that promote motor learning is critical for the design of optimal movement therapies or sports coaching interventions. One possibility, for example, is to provide learners with information in the form of verbal explanations about the to-be-learned motor task. This form of explicit instruction is discussed in different contexts: in the field of movement therapies (McNevin et al. 2000), injury prevention (Benjaminse and Otten 2010) or sports coaching (Hodges and Franks 2002). And although effective instructions might be crucial for learning a motor skill, there is still disagreement about the role of this form of information (Hodges and Franks 2002). Thus, contradictory effects of explicit instructions on the learning of different motor skills have been described. While some studies show positive effects of instructions on learning (Hardy et al. 1996; Prapavessis and McNair 1999; McNair et al. 2000), other studies reveal a negative effect (Wulf and Weigelt 1997; Gredin and Williams 2016).

In the field of sensorimotor adaptation, on the other hand, the state of research is clearer. In this special form of motor learning, already learned movements are adapted to changed environmental conditions. This plays a role, for instance, in the rehabilitation of stroke patients or in the athletic training of adolescents who have to adapt their motor programs due to length growth. Sensorimotor adaptation is studied, for example, by introducing a perturbation between actual movement and visual feedback during simple target reaching movements. In this case, explicit instructions consist of explanations about the nature of the discrepancy between proprioceptive and visual feedback. They clearly lead to improved adaptation (Benson et al. 2011; Werner et al. 2015), especially early in learning (Taylor et al. 2014; Werner et al. 2015; Neville and Cressman 2018; Wang et al. 2019; Bouchard and Cressman 2021). This improvement in initial adaptation is evident even among older participants, although it is not as pronounced here (Vachon et al. 2020). Explicit instructions also improve visuomotor adaptation in cerebellar patients who normally show significant impairment in motor learning (Taylor et al. 2010) and enable simultaneous adaptation to opposing perturbations (dual adaptation) even under conditions where no learning occurs without instructions (Ayala and Henriques 2021). On the other hand, however, explicit instructions about the nature of the perturbation result in reduced aftereffects (Benson et al. 2011; Werner et al. 2015) and have no influence on hand-localization estimates, thus they do not benefit proprioceptive recalibration (Modchalingam et al. 2019). All these results support the idea that explicit instructions lead to the use of cognitive strategies and thus primarily enhance the explicit process and reduce the implicit process of adaptation (Werner et al. 2015; Neville and Cressman 2018). Accordingly, it is not surprising that instructed participants also show a greater transfer of learning to the untrained hand, as recent research shows that this intermanual transfer can be largely related to the explicit process (Poh et al. 2016; Werner et al. 2019; Bouchard and Cressman 2021).

In other studies, instead of explicit instructions on the nature of distorted feedback, participants were provided with a specific and effective strategy to cope with the induced visual perturbation. Specifically, they were instructed to counteract the visual distortion by aiming at the adjacent target point. These participants initially show a large reduction in movement errors and good task performance, but their performance deteriorates again over the longer adaptation period (Mazzoni and Krakauer 2006; Taylor et al. 2010; Rand and Rentsch 2015). The cognitive strategy fails because the implicit process of adaptation occurs simultaneously and is added to the unchanging strategic behavior. This eventually leads to a kind of overlearning. However, this deterioration of performance does not occur if only explicit instructions are given at the beginning of learning instead of a specific strategy. The underlying assumption, which to our knowledge has not been directly investigated, is that here the explicit and implicit processes flexibly come into play. That is, the central nervous system appears to modulate the use of instructions individually and to flexibly adapt the cognitive strategies used to the simultaneous implicit adaptation. However, because instructions have always been given prior to the start of adaptation, it would also be possible for participants to simply “forgot” to use cognitive strategies during the course of adaptation.

In the present study, we therefore investigate the effects of repeated explicit instructions before and during learning on visuomotor adaptation. If we find no overlearning but similar or better adaptation compared to the one-time instruction, the idea of flexible application of explicit and implicit processes is supported. Another goal of our study is to find out whether multiple instructions promote the explicit adaptation process more strongly and thus lead to greater awareness of what is learned and greater intermanual transfer than one-time explicit instructions. In addition, we aim to use a comprehensive study design to confirm previous research findings on the relationship between awareness and intermanual transfer. We will measure adaptation, awareness, intermanual transfer and aftereffects of learning in several conditions: gradual adaptation, sudden adaptation without verbal instructions, sudden adaptation with a one-time instruction before adaptation, and sudden adaptation with several instructions before and during adaptation.

## Method

### Participants

Forty-eight healthy volunteers participated in our study and were randomly divided into four groups of twelve subjects each. All participants were right-handed according to the Edinburgh Handedness Inventory (Oldfield 1971). Participants between the ages of 18 and 30 were recruited and care was taken to ensure that the groups were age-and sex-matched.^1^ None of the participants had any prior experience in visuomotor adaptation research. The authors’ local Ethics Committee had approved the procedure of the experiment, all participants gave written informed consent and the experimental protocol was conducted according to the principles expressed in the Declaration of Helsinki.

### Task

The seated participants watched a computer screen through a mirror, such that the virtual image of the screen coincided with the horizontal surface of a digitizing tablet (see Werner et al. 2019). A starting dot appeared for a duration of 3 s plus a random interval of up to 500 ms in the center of the virtual display, and was then replaced by one of eight possible target dots, located 45° apart along an imaginary circle of 5 cm radius about the center. The target dots were displayed for a duration of 1000 ms. Participants held a digitizing pen in their hand, and reached at each target and back by moving the pen across the digitizing tablet. They were unable to see their arm, due to the mirror and surrounding shrouds; however, pen position was registered and displayed on the screen as a cursor to provide visual feedback about instantaneous hand position. The participants were instructed to reach as quickly and accurately as possible. Reaching movements were performed during episodes of 35 s duration which were interrupted by rest breaks of 5 s. There were about 8 movements per episode.

### Experimental design

All participants were first familiarized with the set-up by performing three episodes under veridical visual feedback, i.e., pen and cursor movements were congruent. Subsequently, participants performed baseline episodes without visual feedback, i.e., no cursor visible, as well as baseline episodes with the left and right hand. In the following adaptation phase of 25 episodes, visuomotor adaptation was induced by rotating the cursor 60° CCW around the central point. Group GNI adapted to the gradually introduced perturbation (increase of 3° per episode, full 60° rotation during the last five episodes) and groups SNI, SOI, and SSI to the suddenly introduced perturbation. While groups GNI and SNI did not receive instructions on the nature of the perturbation, groups SOI and SSI were instructed once before the adaptation block and group SSI was additionally instructed four times during adaptation after every 5th episode. Instructions were given with the help of an illustration of a clock face as in Benson et al. (2011). The adaptation phase was followed by two episodes each of inclusion and exclusion to test for awareness in a process dissociation procedure as in Jacoby (1991) and as previously applied in visuomotor adaptation research (Werner et al. 2015, 2019, 2021; Neville and Cressman 2018; Bouchard and Cressman 2021; Maresch et al. 2021; Ayala and Henriques 2021). Before inclusion, participants were instructed to “use what was learned during adaptation”; and before exclusion, participants were asked to “refrain from using what was learned and to perform movements as during baseline”. No visual feedback was given in those episodes and the order of inclusion and exclusion episodes was randomized between participants. During adaptation and awareness test movements were performed with the right hand. In the following test of intermanual transfer, participants used their left hand and there was no visual feedback to avoid confounding transfer with learning benefits to opposite limb learning (Joiner et al. 2013; Poh et al. 2016). Finally, a five-episode washout phase was performed under veridical feedback and with the right hand. Between all individual tests (awareness, intermanual transfer, washout), two refresh episodes were performed under rotated feedback and also using the right hand. Table 1 shows an overview of the experimental protocol.

**Table 1:** Experimental protocol

Blockname	# of episodes	Visual FB
Familiarization	3	0°
Baseline no FB	2	–
Baseline left hand	2	0°
Baseline right hand	2	0°
Adaptation	25	G or S 60°
Exclusion/inclusion	2	–
Refresh	2	60°
Inclusion/exclusion	2	–
Refresh	2	60°
Intermanual transfer	2	–
Refresh	2	60°
Washout	5	0°

Visual feedback (FB) was either not present (–), veridical (0°) or rotated (60°) gradually (G) or suddenly (S). In the gradual condition rotation size was increased in steps of 3° per episode. The order of exclusion and inclusion was alternated between participants

### Data processing

For each movement and each participant, the direction of reaching movement was determined as the angle between the target direction and the line connecting the hand position at motion onset and the position 150 ms later. Motion onset was determined by a velocity threshold of 30 mm/s. To ensure that only targeted movements were included in the data analysis, several measures were taken: first, all movements were sorted out whose movement amplitude was not yet at least 10 mm away from the movement start at the time the error angle was measured (150 ms). This prevented contamination of the data by small sub-movements at the starting dot that did not represent an intentional, purposeful movement toward the target. Second, all movements with a reaction time greater than 1000 ms, i.e., after the target disappeared, were sorted out. Thus, out of a total of 9427 movements during the adaptation phase, 1267 movements, or about 13%, were removed from the analysis. From single movement data we calculated mean movement directions for each participant and episode. Data are provided as supporting information S1_Dataset and on OSF (https://osf.io/gau3y/). From this, indices for adaptation, intermanual transfer, and washout were calculated for each subject as

$$I = \left( {A - B} \right)/\left( {R - B} \right).$$

Here, *A* is the mean value of all movement directions of each block of five episodes for adaptation or the first episode of intermanual transfer and of washout, respectively. *B* is the mean value of all movement directions of both episodes of the baseline condition with the right (adaptation index and washout index) or with the left hand (intermanual transfer) and *R* is the magnitude of the rotation angle (60°). For each index, the value 1 means complete adaptation, intermanual transfer or washout, while the values − 1 to 0 mean no adaptation, intermanual transfer or washout. Furthermore, we determined the exclusion and inclusion indices for each subject as

$$I = \left( {{\text{EX}} - B_{0} } \right)/\left( {A_{{\text{L}}} - B} \right)\;{\text{and}}\;I = \left( {{\text{IN}} - B_{0} } \right)/\left( {A_{{\text{L}}} - B} \right).$$

Here, EX and IN are the mean values of all movement directions of the first exclusion and inclusion episode, respectively. *B*_0_ is the mean value of all movement directions of both baseline episodes without visual feedback and *A*_L_ is the mean value of all movement directions of the last five adaptation episodes. Following the logic of the process dissociation procedure, performance during the exclusion task should not differ from performance during the baseline if all knowledge acquired through adaptation is conscious. Thus, a small exclusion index indicates a high degree of awareness, and a large exclusion index indicates a greater degree of implicit learning. Within the PDP, awareness can be calculated as the difference between exclusion and inclusion performance. Consequently, we determined the awareness index as inclusion index minus exclusion index (Werner et al. 2015, 2019, 2021). For comparability to other sensorimotor adaptation experiments with different rotation angles, indices were used instead of the simple error angle. However, we recognize that this can be unnecessarily complicated and make interpretation of the data difficult. Therefore, all indices are provided as supporting information S2_Dataset and on OSF (https://osf.io/gau3y/).

For the statistical analysis baseline and adaptation data were submitted to two analyses of variance (ANOVA) with the between-factor Group (GNI, SNI, SOI, SSI) and the within-factor Block. In addition, we submitted the awareness, intermanual transfer, washout and exclusion indices to separate one-factor ANOVAs again with the between-factor Group (GNI, SNI, SOI, SSI). Huynh–Feldt adjustments were applied whenever necessary to compensate for heterogeneity of variances. The effect size is reported for significant differences as Eta-squared *η*^2^. Significant effects were explored with Fisher LSD post hoc tests. The adaptation indices of SOI and SSI of those episodes directly following instruction in SSI were compared using Student's* t*-test for independent samples or, in the case of unequal variances, Welch’s *t*-test. Moreover, partial correlations with the control variable Group (GNI, SNI, SOI, SSI) between awareness index and intermanual transfer, washout, or exclusion index were calculated. The effect size is reported for significant differences as Pearson correlation coefficient R. All these statistical comparisons were performed using SPSS (Version 27.0. Armonk, NY: IBM Corp.).

## Results

### Adaptation

**Fig. 1:**
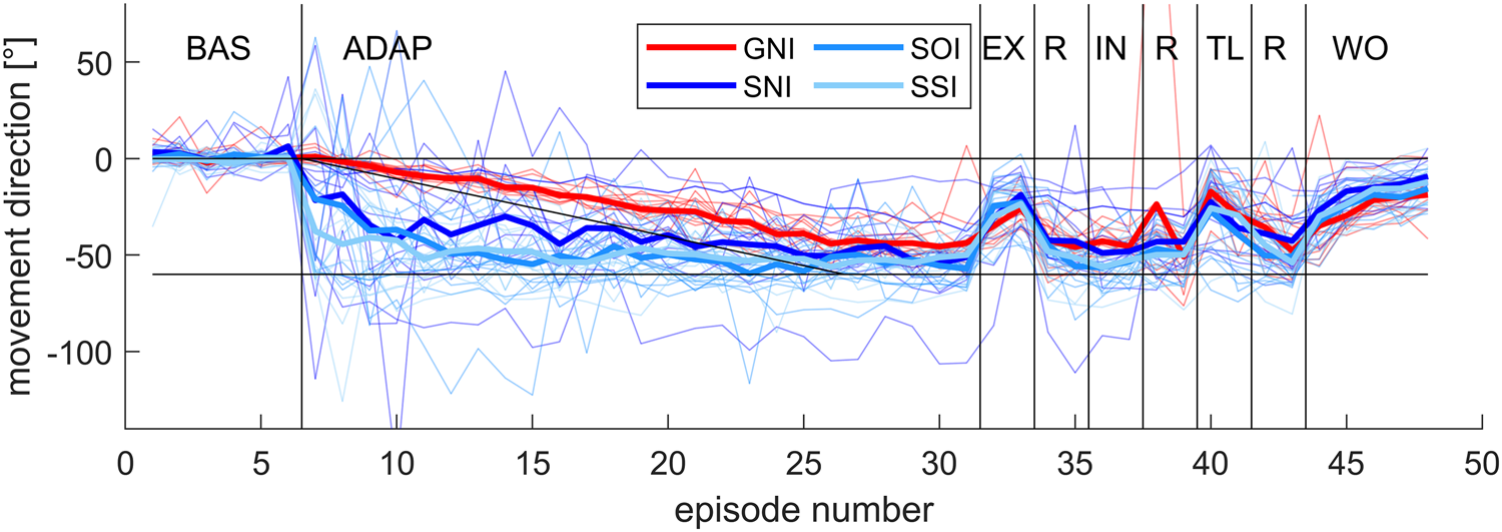
Movement directions of all episodes. Shown are mean movement directions of each episode with respect to target direction of all participants of all groups for baseline (BL), adaptation (ADAP), exclusion (EX), refresh (R), inclusion (IN), transfer to the left hand (TL), and washout (WO) phase. Note that the order of exclusion and inclusion was randomized between participants

Figure 1 shows the mean movement directions of each episode for all experimental phases and each subject. Although mean values of about eight trials each are already shown here, the scatter of the data is very large and outliers are obvious. We decided not to further sort out any data for several reasons. Most importantly, a large variability in movement directions is to be expected, especially in this study on the influence of explicit instruction and related cognitive strategies. This is also supported by the fact that the variability during baseline and final washout phase is much smaller. In addition, with 13% of the movements already excluded, we would rather leave individual outliners in the analysis than potentially exclude further design-related movements. Nevertheless, future studies should test at least 24 participants per group to reduce the effects of outlier trials and outlier participants. During the baseline condition, the movement directions of all participants are around 0°, i.e. the movement errors are very small. During the following adaptation phase, group-specific differences become apparent. While in the gradual group the movement direction increases slowly and steadily up to about − 50°, the movement directions of the three sudden groups are strongly fluctuating, decrease more suddenly and seem to reach a value of around − 50° earlier than in the gradual group. In the following test phases (exclusion and inclusion of the awareness test, intermanual transfer, and washout), the movement directions of the participants in each group are quite similar.

**Fig. 2:**
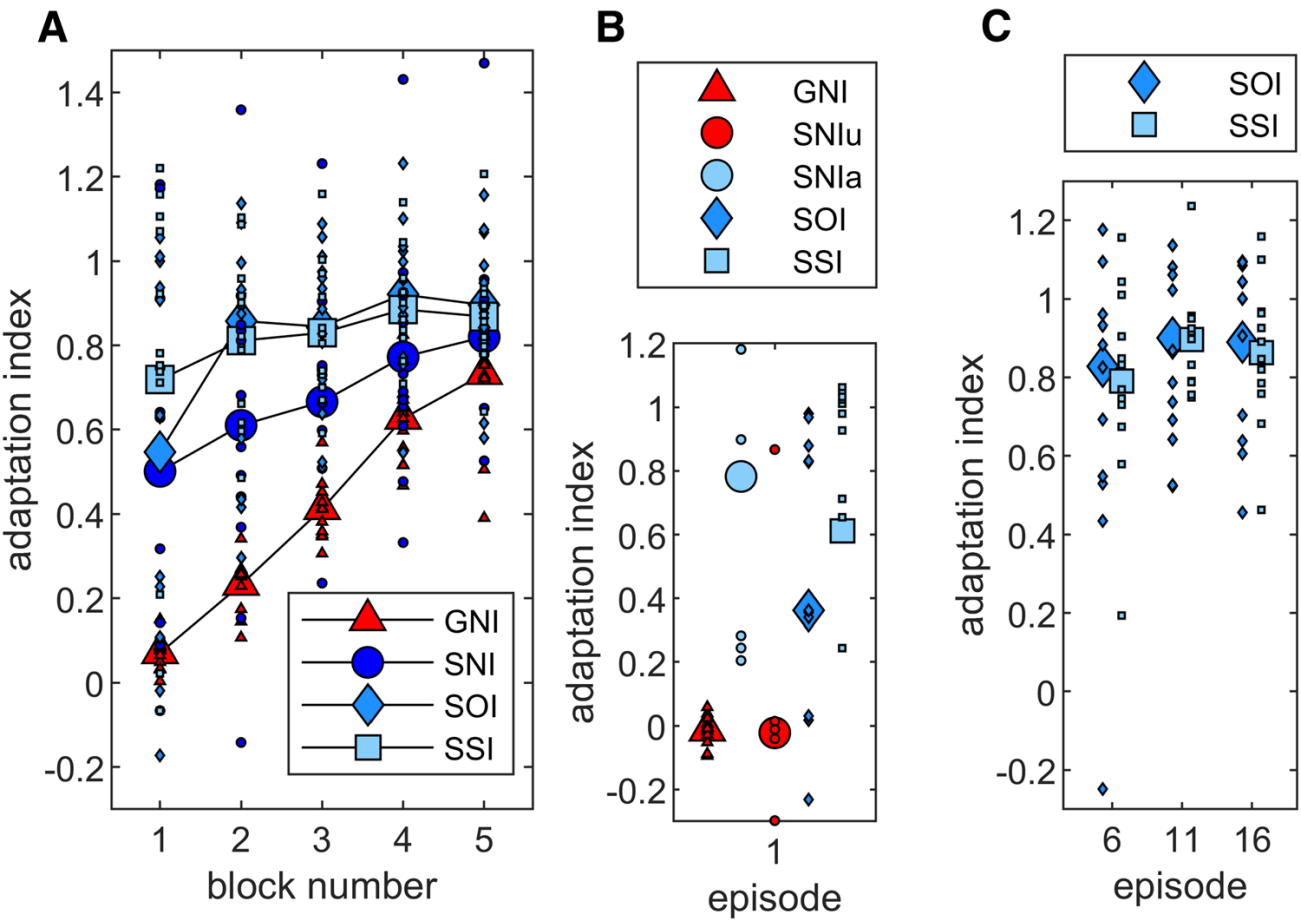
Mean adaptation indices of all blocks and groups. Adaptation index of all blocks for all participants exposed to a gradual rotation (GNI) or a sudden rotation without instructions (SNI), with one-time instruction (SOI) and with several instructions (SSI). All groups are exposed to the full 60° rotation in block 5. Symbols indicate across-subject means and dots represent individual data (**a**). In addition, the adaptation indices of these groups for the first episode are depicted. Instead of a mean index for SNI, we show here separate values for unaware (SNIu) and aware (SNIa) participants of this group (**b**). The third subplot shows the adaptation indices of the 6th, 11th and 16th episodes of the two groups SOI and SSI (**c**)

Figure 2A depicts the mean group adaptation indices for each block of five episodes. Except for the first block, the course of the two instructed groups SOI and SSI is very similar. The figure also shows that these two groups adapt faster than the sudden group without instructions (SNI). All groups reach an index of about 0.8 at the end of the adaptation phase. It should be noted that the adaptation index measures adaptation as a function of the full rotation magnitude of 60°. Therefore the adaptation index of GNI increases only slowly over the course of the adaptation phase, although this group shows very small movement errors throughout adaptation. They also reached the full perturbation in the 21st episode, so all groups were exposed to a 60° rotation of visual feedback during the 5th block.

The above observations of behavior during baseline episodes and adaptation blocks are confirmed by statistical analysis. ANOVA of the movement directions of baseline phase with the factors Group (GNI, SNI, SOI, SSI) and Block (mean of two episodes) yields no significant differences between Groups (*F*(3, 44) = 1.404, *p* = 0.254), Block (*F*(2, 83) = 2.956, *p* = 0.060), or their interaction (*F*(6, 83) = 0.558, *p* = 0.753). The ANOVA of the adaptation index, on the other hand, shows significant differences for all comparisons [Group: *F*(3, 44) = 11.644, *p* < 0.001, *η*^2^ = 0.443; Block: *F*(2, 104) = 26.551, *p* < 0.001, *η*^2^ = 0.376 Group × Block: *F*(7, 104) = 2.958, *p* < 0.01, *η*^2^ = 0.168]. The post hoc analysis of the Group effect shows a smaller adaptation index for GNI than for SNI (*p* < 0.01), SOI (*p* < 0.001) and SSI (*p* < 0.001). Mean group values of SNI are smaller than those of SOI and SSI but the analysis does not reach a significant difference [SOI: *p* = 0.084, SSI: *p* = 0.066]. Furthermore, the adaptation index does not differ between group SOI and SSI (*p* = 0.905). The post hoc analysis of the Group × Block interaction shows three things. First, the adaptation index within the groups GNI, SNI, and SOI increases from block 1 to block 5 [GNI: *p* < 0.001, SNI: *p* < 0.01, SOI: *p* < 0.01, SSI: *p* = 0.175]. This shows that adaptation has occurred. Second, we find no difference between the adaptation indices of all groups for block 5. That is, all groups reach an equal adaptation level at the end of adaptation phase. Third, the individual comparisons show a lower adaptation index for group GNI than all other groups during the first four blocks and a lower index for group SNI than the two instructed groups during blocks 2 and 3 (see Table 2A).

**Table 2:** Results of post hoc comparisons for the adaptation index

	A						B	
	Block 1	Block 2	Block 3	Block 4	Block 5		Episode 1	
	GNI	GNI	GNI	GNI	GNI		SNIu	SNIa
SNI	< 0.01	< 0.01	< 0.01	0.061	0.287	GNI	0.977	< 0.01
SOI	< 0.01	< 0.001	< 0.001	< 0.001	0.050	SOI	0.154	0.121
SSI	< 0.001	< 0.001	< 0.001	< 0.01	0.101	SSI	< 0.05	0.524
	SNI	SNI	SNI	SNI	SNI		SNIa	
SOI	0.768	< 0.05	< 0.05	0.058	0.355	SNIu	< 0.05	
SSI	0.171	0.102	< 0.05	0.145	0.552			
	SOI	SOI	SOI	SOI	SOI			
SSI	0.279	0.698	0.857	0.644	0.738			

The results of the post hoc comparisons between all groups. Shown are the *p* values for the significant Group × Block interaction of the repeated measurement ANOVA of the adaptation indices of all blocks (A) and for the significant Group effect of the one-way ANOVA of the adaptation index of the first episode (B)

One could argue that the different rotational size of GNI during the first four blocks is confounding. Therefore, we repeated the ANOVA with only the SNI, SOI, and SSI groups. The analysis only reveals a significant difference for the factor Block [Group: *F*(2, 33) = 1.709, *p* = 0.197, *η*^2^ = 0.094; Block: *F*(2, 77) = 8.699, *p* < 0.001, *η*^2^ = 0.209; Group × Block: *F*(5, 77) = 0.767, *p* = 0.568, *η*^2^ = 0.044]. In addition, we performed a one-factor analysis of variance with all four groups for block 5 only. In this block, all participants were exposed to the full rotation of 60°. Just as in our comprehensive analysis, the ANOVA yields no effect of group for this block [Group: *F*(3, 47) = 1.557, *p* = 0.213, *η*^2^ = 0.096].

We find no difference in this comprehensive analysis between adaptation after a one-time explicit instruction or after multiple instructions. However, it could be that the repeated instruction has only a short-term effect on the adaptation of the immediately following episode. To find out, we compared the adaptation indices of episodes 6, 11, and 16 of only the two groups SOI and SSI. As can be seen in Fig. 2C, the respective adaptation indices are very similar. Accordingly, t-tests for each episode reveal no significant differences [Episode 6: *t*(22) = 0.215, *p* = 0.832; Episode 11: *t*(14) = 0.040, *p* = 0.969; Episode 16: *t*(22) = 0.302, *p* = 0.765]. Thus, our data show neither a long- nor a short-term positive effect of multiple explicit instructions on adaptation.

### Awareness, intermanual transfer, washout and exclusion

**Fig. 3:**
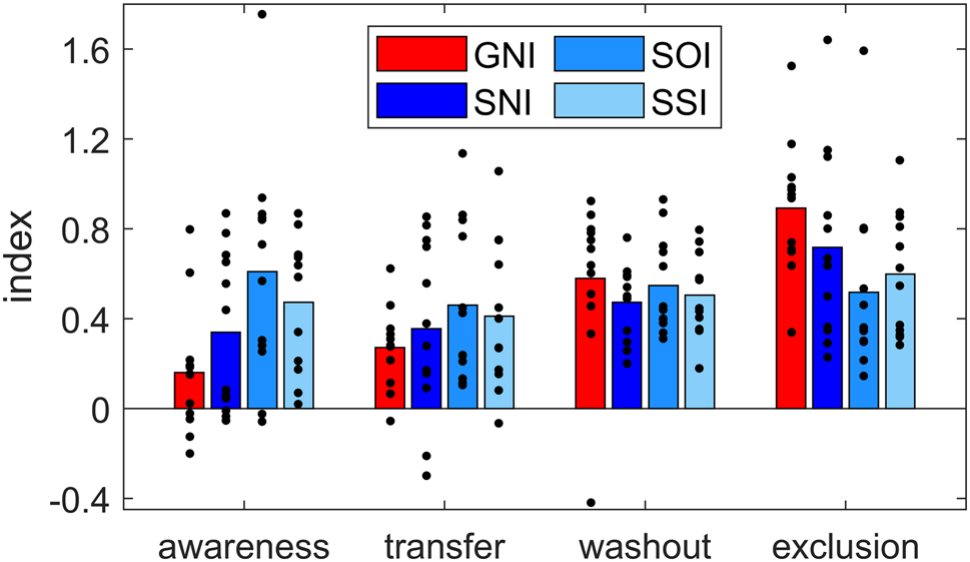
Awareness, intermanual transfer, washout and exclusion. Shown are group mean values for awareness, intermanual transfer, washout and exclusion for all participants exposed to a gradual rotation (GNI) or a sudden rotation without instructions (SNI), with one-time instruction (SOI) and with several instructions (SSI). Note that all indices shown are based on the first episode of the respective phase. Dots represent individual data

**Fig. 4:**
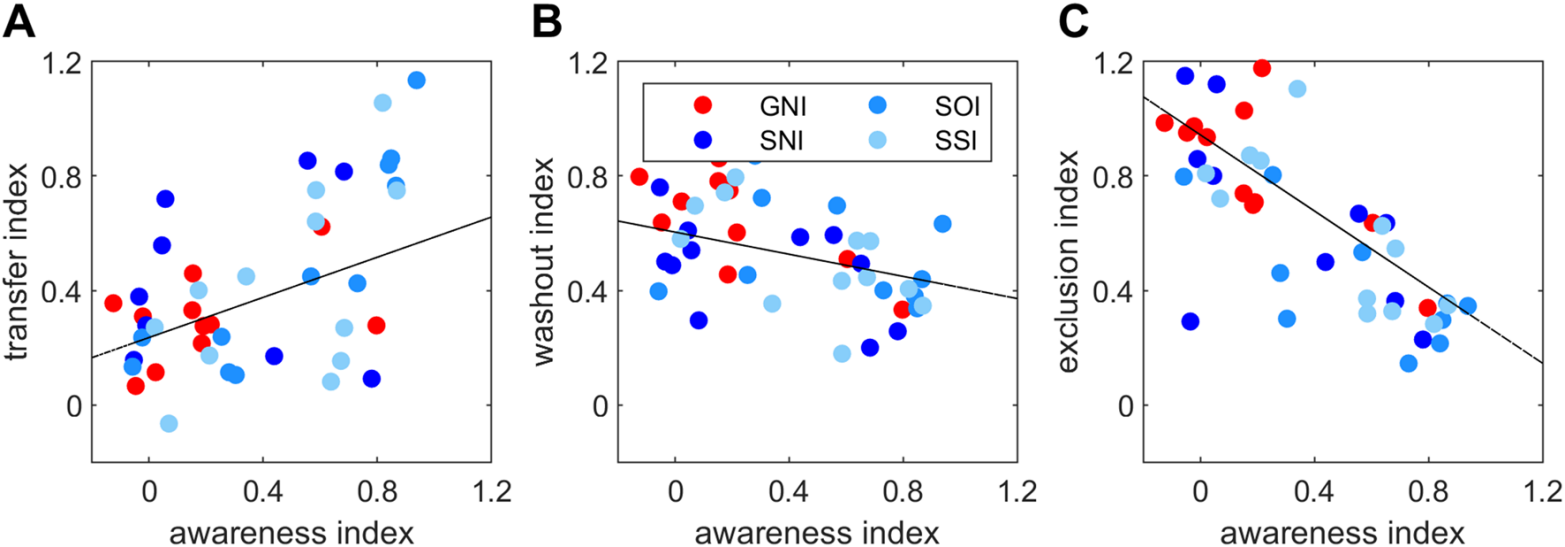
Correlations between awareness, intermanual transfer, washout and exclusion. Correlations between the awareness and transfer (**A**), washout (**B**) or exclusion (**C**) indices for all participants

The results of the awareness, intermanual transfer, washout and exclusion indices are presented in Fig. 3. Note that the washout index shown is based on the first episode of washout phase only. Note also that the exclusion index is calculated as the reaching direction during the first exclusion episode divided by the reaching direction of the last adaptation block, each normalized to the baseline movements. Thus, the more the participant was able to exclude the perturbation, the closer the direction of the reaching movement is to 0° and the smaller the exclusion index. Conversely, the larger the index is, the larger the aftereffect, or the amount of implicit learning. The figure shows that the mean awareness index of the group GNI is lower than that of SNI, and the latter in turn than SOI and SSI. A similar pattern, but not as pronounced, can be seen for the intermanual transfer index. The washout and exclusion index data show an opposite pattern with a (slightly) larger mean index in GNI than in the other groups. The one-way ANOVA with the factor Group (GNI, SNI, SOI, SSI) yields a significant Group effect for the awareness index (*F*(3, 47) = 3.187, *p* < 0.05, *η*^2^ = 0.178). Post hoc analysis shows no difference between GNI and SNI (*p* = 0.246) but a significantly lower value for GNI than SOI (*p* < 0.01) and SSI (*p* < 0.05). Statistical analysis further shows no significant group difference for intermanual transfer (*F*(3, 47) = 0.749, *p* = 0.529), washout (*F*(3, 47) = 0.432, *p* = 0.731) and exclusion indices (*F*(3, 47) = 2.535, *p* = 0.069). In addition, partial correlations with the control variable Group yield a significant correlation between awareness and intermanual transfer index with (*R* = 0.400, *p* < 0.01), with washout index (*R* = − 0.321, *p* < 0.05) and with exclusion index (*R* = − 0.674 *p* < 0.001), respectively. Figure 4A, B, and C shows the corresponding correlations, respectively.

Group SNI adapted to a suddenly introduced perturbation without receiving any instructions. From Fig. 3, it can be seen that the mean awareness index of this group is between that of GNI on the one hand and the indices of SOI and SSI on the other. However, it is striking that part of the subjects (*n* = 6) of SNI show a very low awareness index around the value 0 and another part of the subjects (*n* = 6) show a high awareness index with values between 0.5 and 1. This indicates that half of the subjects were rather unaware regarding the nature of the perturbation while the other half probably independently inferred the nature of the perturbation due to the large movement error. In addition, previous studies show that instructions lead to reduced movement errors, especially at the beginning of adaptation (Benson et al. 2011; Werner et al. 2015). The comparison of our adaptation data of the groups SNI, SOI and SSI, however, reveals a positive effect of instructions on the adaptation index of block 2 and 3 (see Fig. 2A and Table 2A) but not on the adaptation index of the first block. It is possible that explicit instructions lead to increased early adaptation only if the comparison data come from subjects who are actually unaware concerning the nature of the perturbation. Or, in other words, if uninstructed subjects independently recognize the nature of the perturbation, they might show rapid adaptation just as instructed subjects do.

To find out whether the aware and unaware participants of the SNI group actually differed with respect to their initial adaptation index we divided this group depending on their awareness index and performed an additional one-way ANOVA including the groups GNI, SNI_unaware_, SNI_aware_, SOI and SSI on the adaptation index of the first episode. Indeed, this analysis yields a significant group difference (*F*(4, 47) = 3.879, *p* < 0.01, *η*^2^ = 0.265). As can be seen in Fig. 2B and from Table 2B, the adaptation index of SNI_unaware_ is lower than that of SNI_aware_. Moreover, we find significant differences between SNI_unaware_ and SSI and between SNI_aware_ and GNI, respectively. This implies that those participants who independently gained awareness regarding the nature of the perturbation have a similar advantage at the beginning of adaptation as instructed participants. We do not find this effect on the intermanual transfer and washout indices. Here, the statistical analyses do not reveal any significant group differences [intermanual transfer: *F*(4, 47) = 1.043, *p* = 0.396; washout: *F*(4, 47) = 1.565, *p* = 0.201].

## Discussion

The aim of the present study was to determine the effect of multiple explicit instructions on visuomotor adaptation, awareness, and intermanual transfer of learning. In a broad study design, 48 participants adapted to a 60° rotation of visual feedback. Twelve participants were each assigned to one of the following conditions: gradual adaptation (GNI), sudden adaptation without instructions (SNI), sudden adaptation with a one-time instruction (SOI) before adaptation, and sudden adaptation with several instructions (SSI) before and during adaptation. The explicit instructions explained the nature of the perturbation of visual feedback and were given with the help of an illustration of a clock face (Benson et al. 2011).

### Multiple versus one-time instructions

Our results show no improvement in adaptation with multiple explicit instructions compared to a one-time instruction prior to the onset of learning. Neither the analysis with four nor the one with three groups shows a difference here. Also, a close examination of the first adaptation episode after each instruction in SSI does not show a larger adaptation index in SSI than in SOI. Thus, the effect of explicit instructions is not enhanced by repetition—neither in the short nor in the long run. Accordingly, while it is common in sports training to repeat instructions, this does not seem to provide a learning advantage in sensorimotor adaptation. On the other hand, we also find no deterioration in adaptation from multiple instructions, as is the case when using a specific cognitive strategy (Mazzoni and Krakauer 2006; Taylor et al. 2010; Rand and Rentsch 2015). Moreover, we find no difference in awareness, intermanual transfer or exclusion between the two groups SOI and SSI. In summary, these results can be interpreted as follows: first, multiple instructions do not seem to further increase the size of the explicit process. We would otherwise need to find greater awareness in SSI than SOI (Bouchard and Cressman 2021). Second, we find no evidence that the one-time instruction was "forgotten". We would otherwise have to find lower awareness in SOI than SSI. This result is also consistent with a previous finding that the size of the explicit process after a single explicit instruction remains the same throughout the adaptation phase (Neville and Cressman 2018). Third, the data suggest that the central nervous system applies cognitive strategies flexibly and accounts for concurrent implicit adaptation. We would otherwise have to find a deterioration of adaptation with multiple instructions (Mazzoni and Krakauer 2006; Taylor et al. 2010; Rand and Rentsch 2015).

One might argue that the participants are not very likely to forget to use cognitive strategies during adaptation since the instructions are given immediately before the adaptation phase. Perhaps a control group receiving instructions at the beginning of the experiment (before baseline) and another receiving repeated instructions during baseline could complete the picture and give a better understanding of the importance of instructions timing versus repetitions. Future research should be conducted to scrutinize this possibility.

### Instructions versus no instructions

Our data reveal a trend for greater general adaptation among instructed participants (SOI and SSI) than among uninstructed participants (SNI). Examination of the individual adaptation blocks shows that this difference is more pronounced at the beginning of the adaptation phase. This is consistent with the results of previous studies (Benson et al. 2011; Taylor et al. 2014; Werner et al. 2015; Neville and Cressman 2018; Wang et al. 2019; Bouchard and Cressman 2021). However, our reduced analysis with the three groups SNI, SOI and SSI cannot confirm this difference. While it is true that the difference in rotation size during the first four blocks in GNI probably had a confounding effect, we wanted to broadly manipulate the level of awareness in a comprehensive study design. The extreme conditions of no awareness during gradual adaptation to (presumably) very large awareness after repeated instructions present an interesting comparison. Moreover, the comparison of gradual and sudden adaptation is simply not possible without the difference in rotation size and has therefore already been made in different studies despite this limitation (Klassen et al. 2005; Galea et al. 2010; Wang et al. 2011).

In the present experiment, we provided explicit explanations about the nature of the visual perturbation without examining the resulting consequences for the participants in more detail. However, previous studies show enlarged reaction times (Benson et al. 2011) and prefrontal cortex involvement (Anguera et al. 2010) in instructed subjects. Taken together, these results suggest that the instructed participants used more cognitive strategies. This may also be true for some participants in the non-instructed (SNI) group. This is because our data reveal, for the first time, that those participants who independently develop an awareness of the nature of the perturbation show exactly the same improvements in adaptation as the instructed participants. Similar results were also found in an experiment on visuomotor sequence learning: participants who discovered the rules spontaneously showed similar behavior to participants who were instructed. Both showed fewer errors in a transfer session than unaware participants (Tanaka and Watanabe 2017). In previous studies, explicit instruction about the nature of the perturbation did lead to improved adaptation, but this was at the expense of reduced aftereffects of visuomotor (Benson et al. 2011; Werner et al. 2015) or locomotor learning (French et al. 2018). Accordingly, while we find no group differences for the washout and exclusion indices between instructed and non-instructed participants, we do find a negative correlation between the amount of awareness gained during adaptation and washout (rather shallow slope) and exclusion.

Since we provided visual feedback during the washout phase in the present experiment, our exclusion index is the parameter that can be most closely compared to the aftereffect commonly found in the literature. Consistent with previous results (Kagerer et al. 1997; Ingram et al. 2000; Michel et al. 2007; Werner and Bock 2007; Benson et al. 2011; Wong and Shelhamer 2011; Werner et al. 2015), we see a larger mean aftereffect in the gradual and non-instructed groups, although statistical analysis here only reveals a trend for a difference (*p* = 0.069). The size of our exclusion index is similar to an early study (Maresch et al. 2021) but slightly larger than aftereffects in some other studies (Salomonczyk et al. 2012; Werner et al. 2015; Bond and Taylor 2015). We suspect that differences in study design contribute to these variations in aftereffect size. For example, some studies have examined adaptation to smaller rotations of only 30° (Salomonczyk et al. 2012) or 45° (Bond and Taylor 2015).

### Intermanual transfer

Another aim of the present study was to examine the relationship between awareness and intermanual transfer in a comprehensive study design. Consistent with previous research we found greater intermanual transfer in more aware participants across all groups. Our data thus confirm the idea that transfer of learning to the other hand is largely related to the explicit process of adaptation (Poh et al. 2016; Werner et al. 2019; Bouchard and Cressman 2021). This is also consistent with recent research showing that the fast adaptation process (Smith et al. 2006) previously associated with the explicit process (McDougle et al. 2015) is responsible for the generalization of learning (Xing and Saunders 2021). In the more detailed analysis of the individual groups, we find a difference in awareness between the gradual and the sudden (especially the instructed) groups, but we do not find a group effect of intermanual transfer. A possible explanation arises from the results of our previous study, in which we showed that a different intermanual transfer after gradual and sudden adaptation occurs only when adapting to a larger rotation (e.g., 75°, Werner et al. 2019). This is because during adaptation to smaller rotation angles (e.g., 30°), participants in the sudden group are just as unaware as those in the gradual group. This could also be the case for a 60° rotation angle. However, the awareness of group SNI in the present study is clearly pronounced with a mean awareness index of 0.34 and is even greater than after sudden adaptation to a 75° rotation with an index of about 0.3 (Werner et al. 2019). An alternative explanation is based on the fact that the amount of transfer cannot be fully explained by awareness of what is learned. There seems to be both an implicit and an explicit component of intermanual transfer (Poh et al. 2016; Werner et al. 2021). In our data, we cannot distinguish these two components. It is possible that in the GNI group the proportion of implicit intermanual transfer is particularly pronounced, for example, due to martial arts training, greater general athletic activity (Werner et al. 2021) or stronger right-handedness (Lefumat et al. 2015; Werner et al. 2021).

The intermanual transfer data, as well as the rest of our data, show very high variability and numerous outliers. As mentioned in the results section, increased variability in the data is to be expected, particularly in studies of the influence of explicit instructions and associated cognitive strategies. Testing at least 24 participants per group would have reduced the effects of outliers and improved the power of the data. The small number of participants should be taken into account when interpreting the results.

## Conclusion

Our results show neither an improvement nor a deterioration of adaptation when multiple explicit instructions are given compared to a single instruction before learning begins. We conclude that the size of the explicit process may not further be increased by instructions and that the central nervous system can flexibly apply cognitive strategies and account for simultaneous implicit adaptation. In addition, we find a positive relationship between awareness of what has been learned and intermanual transfer. The transfer of learning to the other hand thus appears to be largely related to the explicit adaptation process.

## Supplementary Information

Below is the link to the electronic supplementary material.

Supplementary file1 (CSV 34 KB)

Supplementary file2 (CSV 13 KB)

## Data Availability

All data are available in the online repository (https://osf.io/gau3y/).

## Footnotes

1 The handedness questionnaires as well as the forms with the personal data of the participants were unfortunately destroyed in the course of a move to a new institute building. Therefore, it is not possible for us to give the exact number of men and women as well as the exact age of the participants.
